# Supplementary material for: The Systems Biology Research Tool: evolvable open-source software
Source: BMC Syst Biol. 2008 Jun 29;2:55. doi: 10.1186/1752-0509-2-55 (PMC2446383; doi:10.1186/1752-0509-2-55)
Supplement: Additional file 1 — SBRT Archive. An archive of the current version of the Systems Biology Research Tool. [file 1752-0509-2-55-S1.zip › sbrt-1.4.0/doc/developers_guide/api/sbrt/shell/mng/utilities/package-summary.html]

sbrt.shell.mng.utilities


|  |  |  |
| --- | --- | --- |
| **Overview** \| **Package** \| Class \| **Use** \| **Tree** \| **Deprecated** \| **Index** \| **Help** | |  |
| **PREV PACKAGE**   **NEXT PACKAGE** | **FRAMES**    **NO FRAMES**     **All Classes** |  |


---

## Package sbrt.shell.mng.utilities

Provides classes and interfaces for creating mathematical
process managers.

**See:**
  
          **Description**

| **Class Summary** | |
| --- | --- |
| **IntervalComparisonManager** | This class is used to compare the intervals contained in separate files. |
| **VariableParticipationManager** | This class is used to manage variable participation experiments. |
| **VectorComparisonManager** | This class is used to compare the intervals contained in separate files. |

## Package sbrt.shell.mng.utilities Description

Provides classes and interfaces for creating mathematical
process managers.

---


|  |  |  |
| --- | --- | --- |
| **Overview** \| **Package** \| Class \| **Use** \| **Tree** \| **Deprecated** \| **Index** \| **Help** | |  |
| **PREV PACKAGE**   **NEXT PACKAGE** | **FRAMES**    **NO FRAMES**     **All Classes** |  |


---
